# Prescription Behaviour and Drug Interactions of Acid Suppressants With Tyrosine Kinase Inhibitors in Patients With Leukaemia: Data From Germany

**DOI:** 10.1002/jha2.70279

**Published:** 2026-03-28

**Authors:** Marek Lapka, Anna Khryakova, Anastasiia Nazmutdinova, Chrysostomos Charalambous, Jan Bosak, Jiri Sliva

**Affiliations:** ^1^ Department of Pharmacology Third Faculty of Medicine Charles University Prague Czech Republic; ^2^ Department of Medical Affairs Zentiva Group, a.s. Prague Czech Republic

**Keywords:** absorption, acid suppressants, antacids, chronic myeloid leukaemia, drug–drug interaction, H2 antagonists, proton pump inhibitors, tyrosine kinase inhibitors

## Abstract

**Background:**

Tyrosine kinase inhibitors (TKIs) are essential in treating chronic myeloid leukaemia (CML) and other malignancies. Their absorption, however, is highly pH‐dependent, making them susceptible to interactions with acid‐suppressing agents such as proton pump inhibitors (PPIs), histamine‐2 receptor antagonists (H2RAs) and antacids. Despite clear warnings, real‐world data show frequent co‐administration, which may compromise therapeutic efficacy.

**Methods:**

This study combines two approaches—(1) a systematic review summarizing the impact of acid suppressants on TKI pharmacokinetics and clinical outcomes, and (2) an analysis of German prescription data from the Insight Health co‐prescription database, focusing on TKIs primarily used for CML.

**Results:**

Results indicate substantial co‐prescription rates involving dasatinib, nilotinib, imatinib, bosutinib and ponatinib, with variations across years and agents. Pharmacokinetic evidence demonstrates that PPIs and H2RAs can significantly reduce systemic TKI exposure, with reported decreases of up to 96% in peak plasma concentration and 88% in overall exposure. These reductions have been associated with poorer clinical outcomes, including decreased survival.

**Conclusion:**

The findings highlight the need for improved prescriber awareness, adherence to guidelines and risk mitigation strategies such as therapeutic drug monitoring or alternative dosing. Collaborative efforts between clinicians and regulatory bodies are essential to minimize risks and optimize patient outcomes.

## 1 Introduction

Tyrosine kinase (TK) inhibitors (TKIs) are small molecules with demonstrated antitumor activity in various malignancies. TKIs offer the advantage of oral bioavailability and can inhibit multiple TKs involved in tumour growth and angiogenesis, affecting key mediators of cell proliferation and overall survival (OS) [1]. Examples of antiangiogenic TKIs include dasatinib, sorafenib, sunitinib, pazopanib and axitinib, among others. The clinical efficacy of these small‐molecule TKIs is continuously being evaluated in conditions such as chronic myeloid leukaemia (CML), advanced renal cell carcinoma, hepatocellular carcinoma, differentiated thyroid carcinoma, pancreatic neuroendocrine tumours, gastrointestinal stromal tumours and non‐small cell lung cancer [2].

Acid suppressants (A02 according to ATC classification) are a group of drugs that prevent the formation of ulcers or promote the healing of ulcers and include a variety of different drug classes. Histamine‐2 receptor antagonists (H2RAs) (e.g., famotidine) inhibit acid secretion by blocking H2 receptors on the parietal cell [3]. Antacids typically contain a combination of magnesium hydroxide, aluminium hydroxide, calcium carbonate, sodium hydroxycarbonate, alginic acid or others. These compounds neutralize gastric acid, thereby reducing acidity in the stomach and decreasing acid exposure to the duodenum [4]. Lastly, proton pump inhibitors (PPIs) such as omeprazole, lansoprazole, dexlansoprazole, rabeprazole, pantoprazole or esomeprazole block acid secretion by irreversibly binding to and inhibiting the hydrogen‐potassium ATPase pump located on the luminal surface of the parietal cell membrane [5]. For this article's purposes, we will use all the above‐mentioned groups as acid‐suppressants (AS) using this general term. We excluded vonoprazan and similar drugs as they are not well established in the current practice.

Despite the benefits of both drug classes, there is some risk in concomitant administration due to drug–drug interaction (DDI). The absorption of many of the aforementioned TKIs is pH‐dependent, and co‐administration with acid‐suppressing agents may reduce their bioavailability, potentially compromising clinical outcomes [6, 7, 8, 9, 10, 11, 12, 13, 14, 15, 16, 17, 18, 19, 20, 21, 22, 23, 24, 25, 26, 27, 28, 29, 30, 31, 32, 33, 34, 35]. Another study described that nearly one in four older adults with this combination is associated with an increased risk of death [28]. In addition, a systematic review and meta‐analysis of 16 retrospective studies involving 372,418 patients found that this DDI negatively impacts survival outcomes in patients with solid tumours. The pooled hazard ratios for OS and progression‐free survival (PFS) were significantly worse in patients exposed to the DDI compared to those without it [36].

Pharmacokinetic (PK) studies in both healthy volunteers [28, 34, 37, 38, 39, 40, 41, 42, 43, 44, 45, 46, 47, 48, 49, 50, 51, 52, 53, 54, 55, 56, 57] and oncology patients [29, 53, 58, 59, 60, 61] have confirmed that co‐administration of certain TKIs with AS significantly alters the area under the curve (AUC) and/or maximum concentration (C_max_), potentially impacting the drug efficacy.

Despite the well‐documented risks of DDIs in the scientific literature and the explicit warnings in the Summary of Product Characteristics (SmPC) of individual medicinal products (as detailed in Supporting Information), some practitioners continue to prescribe these combinations, either unaware of or disregarding the potential risk of treatment failure.

This article aims to present current evidence on DDIs across the entire TKI class based on scientific literature and to analyse prescription habits in Germany using Insight Health co‐prescription data on administration of TKIs typically used in the treatment of any phase of CML.

## 2 Methods

### 2.1 Search Strategy

The review section of this paper is based on a comprehensive literature search to identify clinically relevant publications focusing on the interaction between acid‐modifying agents, such as antacids, H2RAs and PPIs, with TKIs regardless of their indication. As described in the Section 1, vonoprazan and similar compounds were excluded from the search. Moreover, articles dealing with other drugs than TKIs were not included. Publications indexed in PubMed/MEDLINE, Embase and Cochrane Library were screened in the search performed in June 2025, and the identified publications were assessed for relevance till May 2025.

Detailed search strategy for clinical research can be found in the Supporting Information. The search results are provided in the Supporting Information. Additional articles may have been identified during the manual review of the publications.

### 2.2 Eligibility Criteria

For the assessment of relevance, the following PICOS (population, intervention, comparison, outcome and study design) criteria were applied. The population (P) included adults/elderly or children human participants, either healthy or patients. The intervention (I) consisted of any TKI compound in combination with a PPI/H2RA or antacid. The comparator (C) was any form of control, including a placebo or active treatment or no control. The outcome (O) focused on the measures of (i) PK parameters or (ii) clinically relevant outcomes. The study design (S) included clinical trials. Further exclusion criteria were also applied, such as language (e.g., not English) and types of publications (e.g., reviews, chemical analyses or similar). Publications classified as not relevant or not adhering to the defined criteria were excluded and are not cited in this review.

### 2.3 Insight Health Co‐Prescription Database

Co‐prescription data were obtained via the Insight Health co‐prescription database. This database covers 77% of the German statutory health insurance (SHI) prescriptions from the patient view, with data from more than 64 million patients since March 2020 from all 16 federal states of Germany (https://www.insight‐health.de/deu_en). These data give valid representation of the SHI prescription market at the patient level. Prescribing doctors are divided into 21 specialist groups, including internist subgroups. Unless otherwise specified, co‐prescription data was obtained for the entire year of 2024. A medication was considered as co‐prescribed along with the given TKI if the prescription happened within −15 to +45 days of the TKI prescription. The studied medications that are known to be typically linked to changes in stomach were either part of the entire A02 ATC class or specified to the following categories of A02A (Antacids, antiflatulents, carnatives), A02B1 (H2RAs) and A02B2 (PPIs), or directly to the particular active pharmaceutical ingredient.

Sigmaplot version 11 was used for data visualization of the SHI prescription data.

### 2.4 Current German Labelling

To check the wording in currently available SmPCs of TKIs registered in Germany (Supporting Information), we used publicly accessible website www.dimdi.de during June 2025, where the English versions were retrieved if possible. If not, the German versions were translated.

An overview of SmPC wordings covering the specific DDI of the medicinal products in CML/ALL indication available on the German market (as of January 2025) is presented in Table 1.

**TABLE 1:** Wordings covering the specific DDI in SmPC of the medicinal products in CML/ALL indication available on the German market (as of January 2025).

INN	DDI contraindicated (Section 4.3)	DDI in warnings (Section 4.3)	DDI wording in Section 4.5	
			Interaction with H2 antagonist and PPI	Interaction with antacids
Dasatinib	No	Yes, the concomitant use of dasatinib and a histamine‐2 (H2) antagonist (e.g., famotidine), proton pump inhibitor (e.g., omeprazole), or aluminium hydroxide/magnesium hydroxide may reduce the exposure to dasatinib. Thus, H2 antagonists and proton pump inhibitors are not recommended and aluminium hydroxide/magnesium hydroxide products should be administered up to 2 h prior to, or 2 h following the administration of dasatinib (see Section 4.5).	Yes, in a study of 14 healthy subjects, administration of a single 100‐ mg dose of dasatinib 22 h following a 4‐day, 40‐mg omeprazole dose at steady state reduced the AUC of dasatinib by 43% and the Cmax of dasatinib by 42%. The use of antacids should be considered in place of H2 antagonists or proton pump inhibitors in patients receiving dasatinib therapy (see Section 4.4).	Yes, non‐clinical data demonstrate that the solubility of dasatinib is pH‐dependent. In healthy subjects, the concomitant use of aluminium hydroxide/magnesium hydroxide antacids with dasatinib reduced the AUC of a single dose of dasatinib by 55% and the Cmax by 58%. However, when antacids were administered 2 h prior to a single dose of dasatinib, no relevant changes in dasatinib concentration or exposure were observed. Thus, antacids may be administered up to 2 h prior to or 2 h following dasatinib (see Section 4.4).
Nilotinib	No	No	Yes, nilotinib has pH‐dependent solubility, with lower solubility at higher pH. In healthy subjects receiving esomeprazole at 40 mg once daily for 5 days, gastric pH was markedly increased, but nilotinib absorption was only decreased modestly (27% decrease in Cmax and 34% decrease in AUC0‐∞). Nilotinib may be used concurrently with esomeprazole or other proton pump inhibitors as needed. In a study in healthy subjects, no significant change in nilotinib pharmacokinetics was observed when a single 400 mg dose of nilotinib was administered 10 h after and 2 h before famotidine. Therefore, when the concurrent use of an H2 blocker is necessary, it may be administered approximately 10 h before and approximately 2 h after the dose of nilotinib.	Yes, in the same study as above, administration of an antacid (aluminium hydroxide/magnesium hydroxide/simethicone) 2 h before or after a single 400 mg dose of nilotinib also did not alter nilotinib pharmacokinetics. Therefore, if necessary, an antacid may be administered approximately 2 h before or approximately 2 h after the dose of nilotinib.
Imatinib	No	No	Not applicable	
Bosutinib	No	No	Caution should be exercised when administering bosutinib concomitantly with PPIs. Short‐acting antacids should be considered as an alternative to PPIs, and administration times of bosutinib and antacids should be separated (i.e., take bosutinib in the morning and antacids in the evening) whenever possible. Bosutinib displays pH‐dependent aqueous solubility in vitro. When a single oral dose of bosutinib (400 mg) was co‐administered with multiple oral doses of lansoprazole (60 mg) in a study of 24 healthy fasting subjects, bosutinib Cmax and AUC decreased to 54% and 74%, respectively, of the values seen when bosutinib (400 mg) was given alone.	
Ponatinib	No	No	Not applicable	

## 3 Results

### 3.1 Results of the Literature Search

Considering the literature search, from total 2015 searched articles, 85 were retrieved for relevance to this topic and 61 articles were included for further presentation. The identified studies covered two main evidence domains: PK outcomes and clinical outcomes, including survival or treatment efficacy, as detailed in Tables S1–S3. However, due to substantial heterogeneity in study designs, endpoints, dosing regimens and molecular subgroups, a direct pooling or uniform quantitative synthesis was not feasible. Therefore, individual study‐level results are provided in the Supporting Information, while their structured interpretation is summarized in the Section 4.

These are presented in Tables S1–S3.

### 3.2 Co‐Prescription Trends in Germany

#### 3.2.1 Co‐Prescription of A02 Medications With Various TKIs in CML Treatment

**FIGURE 1:**
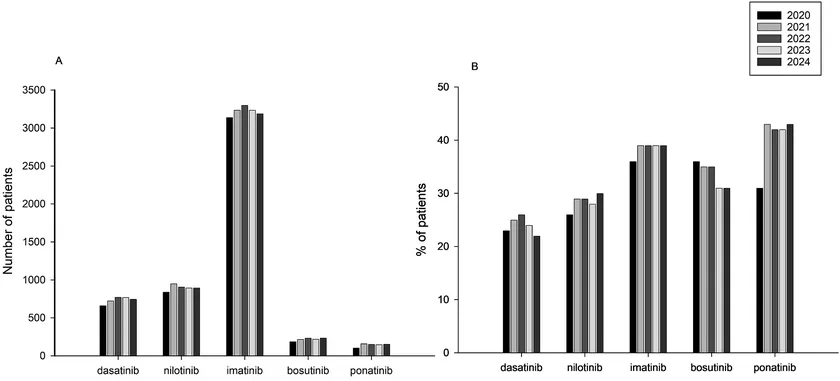
Number (A) and % (B) of TKI patients on A02 molecules in Germany between 2020 and 2024 in CML indication. *Source*: SHI patients, January 2020–December 2024, Patient Insights Analytics, Insight Health.

As shown in the raw data in the Supporting Information, the co‐prescription of A02 medications alongside TKIs used primarily in CML revealed distinct prescription patterns for individual TKIs from 2020 to early 2024. These prescription trends are illustrated in Figure 1A,B.

The proportion of patients receiving A02 drugs alongside specific TKIs was notable and distributed as follows: between years 2020 and 2024, the percentage of patients with co‐administration compared to the overall patient population for dasatinib oscillated between 22% (*n* = 747) and 26% (*n* = 744), for nilotinib between 26% (*n* = 841) and 30% (*n* = 897), for imatinib between 36% (*n* = 3138) and 39% (*n* = 3300), for bosutinib between 30% (*n* = 223) and 36% (*n* = 190) and for ponatinib between 31% (*n* = 106) and 43% (n = 162). Compared to 2020, in 2021 the trend shifted slightly, with increases observed in all groups except bosutinib: dasatinib (24%; *n* = 771), nilotinib (29%; *n* = 951), imatinib (39%; *n* = 3237), bosutinib (35%; *n* = 219) and ponatinib (43%; *n* = 162). By 2022, most percentages remained stable, though a slight increase in A02 prescriptions was observed with imatinib (39%; n = 3300) and ponatinib (42%; *n* = 154). In 2023, a marginal decline was seen with dasatinib (24%; *n* = 771), nilotinib (28%; *n* = 897) and bosutinib (31%; *n* = 223), while imatinib and ponatinib remained somewhat stable. By 2024, some reduction in A02 prescriptions was observed for dasatinib (22%; *n* = 747) and imatinib (39%; *n* = 3189).

##### 3.2.1.1 Demography—Gender Analysis

The demographic distribution of patients prescribed TKIs, along with A02 co‐prescriptions, shows variations by sex and specific TKI molecules. The tabulated data (provided in the Supporting Information) categorizes patients as male, female, or of unknown sex, showing the percentage of those co‐prescribed A02 medications relative to the total patient count in each group.

**FIGURE 2:**
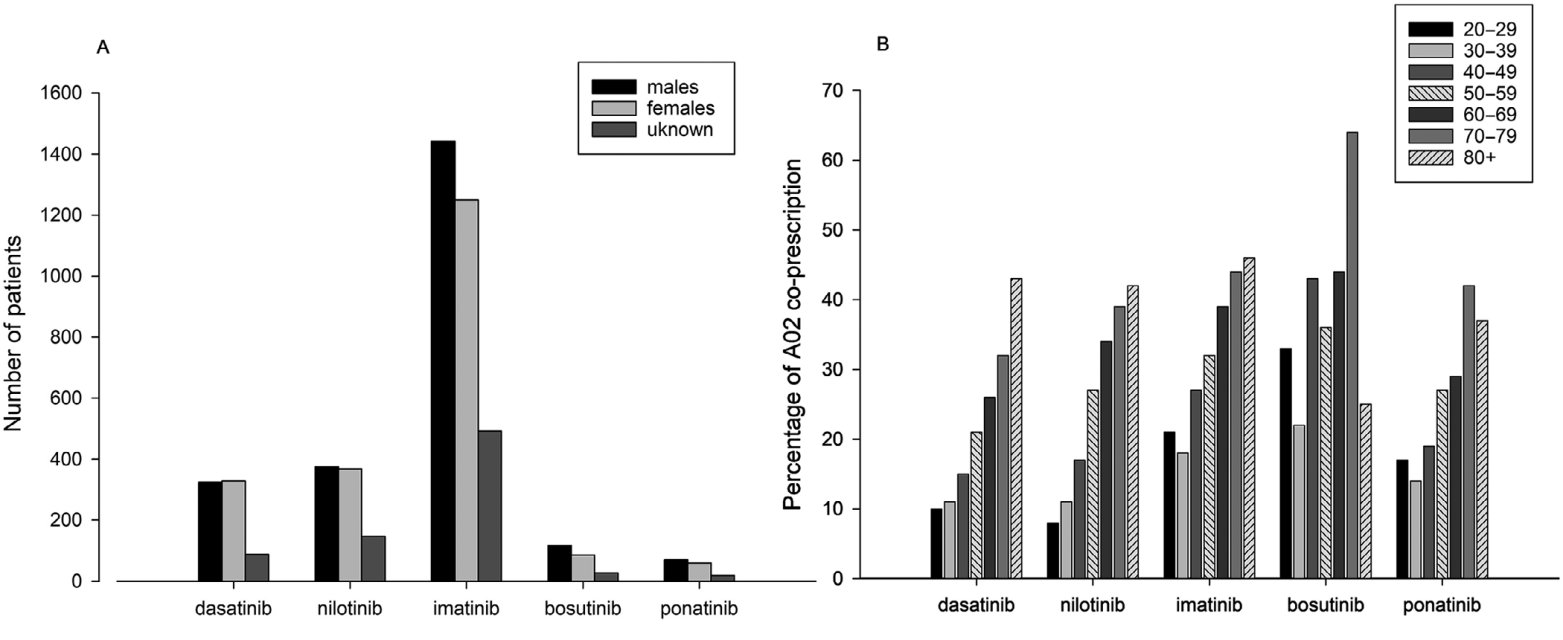
Number of patients using the TKI and PPI combination divided by gender (A) and age (B). *Source*: SHI patients, January 2024–December 2024, Patient Insights Analytics, Insight Health.

As shown in Figure 2A, 326 of 1613 male patients (20.21%) prescribed dasatinib also received A02 medications. Among females, the co‐prescription rate was slightly higher (330 of 1254; 26.32%). For patients of unknown sex, the rate was 19.35% (90 of 465). For nilotinib, 29.70% (376 of 1266) of males and 30.15% (370 of 1227) of females were co‐prescribed A02 drugs, while patients of unknown sex had an intermediate rate of 29.45% (149 of 506). Imatinib exhibited the highest A02 co‐prescription rates across all demographic groups. Among males, 37.60% (1443 of 3838) received A02 medications, with an even higher rate among females at 40.37% (1251 of 3099). Patients of unknown sex had a rate of 40.54% (493 of 1216). For bosutinib, 32.78% (119 of 363) of males were prescribed A02 medications, compared to a higher rate of 30.24% (88 of 291) among females. Patients of unknown sex had a co‐prescription rate of 28.71% (29 of 101). Ponatinib showed the highest A02 co‐prescription rates among all TKIs, with 41.48% (73 of 176) of male patients, 46.97% (62 of 132) of females and 36.84% (21 of 57) of patients of unknown sex receiving A02 medications. As with other TKIs, A02 co‐prescription was more common among females than males. These findings indicate a consistent trend of higher A02 co‐prescription rates among females across all TKIs.

##### 3.2.1.2 Demography—Age Analysis

The age distribution of patients prescribed TKIs shows a clear trend toward increasing use with older age across most agents. Dasatinib and nilotinib demonstrate a gradual rise in prescription rates, starting at 10%–11% in the 20–39 age groups and reaching 43% and 42%, respectively, in patients aged 80 and older. Imatinib follows a similar pattern, with steady increases from 21% in younger patients to 46% in the oldest group, reflecting its well‐established role and tolerability across age categories. Ponatinib presents a distinct pattern. Its use is relatively high in middle‐aged and older adults, peaking at 64% in the 70–79 group, but then declining to 25% in those over 80. This likely reflects its indication for more resistant disease, which may limit its use in very elderly or frail patients. Bosutinib also shows increasing use with age, from 17% in younger patients to 42% and 37% in the 70–79 and 80+ categories, suggesting its growing role in older populations needing alternative TKI options.

As shown in Figure 2B, the data indicate that most prescriptions are concentrated in patients aged 50 and older. Imatinib stands out due to its broad applicability and large cohort size. Younger patients represent a minimal proportion, reflecting the rarity of CML diagnoses in paediatric and adolescent populations [62].

Figure 2B is for better visibility, divided into 10‐year groups and group 0–19 is neglected.

##### 3.2.1.3 Physicians/ Prescribing Specialist (2023)

**FIGURE 3:**
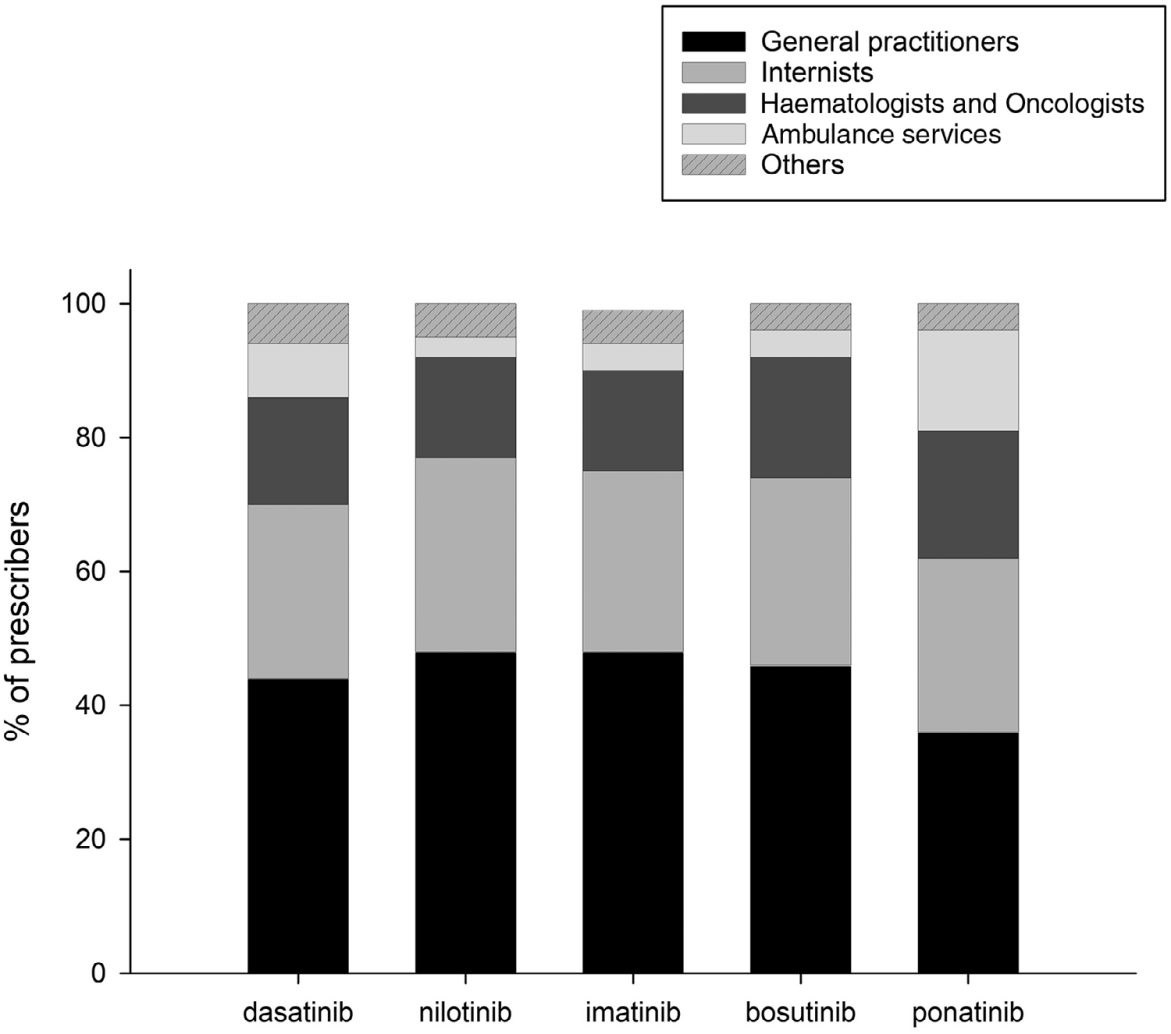
Physician specializations prescribing the A02 product for TKI patients in Germany. *Source*: Prescribing specialization, SHI patients, January 2024–December 2024, Patient Insights Analytics, Insight Health.

The prescribing patterns of TKIs, as shown in Figure 3, reveal notable differences among physician specialities, including general practitioners (GPs), internists, haematologists/oncologists, ambulance services and others.

General practitioners were responsible for 44% of dasatinib prescriptions, reflecting their key role in managing initial treatments or comorbid conditions in CML patients. Internists accounted for 26% of prescriptions, often serving as intermediaries in monitoring long‐term care. Haematologists/oncologists contributed 16%, while ambulance services represented 8% of prescriptions, likely reflecting emergency care or acute interventions. Nilotinib similarly demonstrated a higher reliance on similar trends, with GPs, who also accounted for 48% of prescriptions. Internists contributed 29%, similar to their involvement with dasatinib, while haematologists/oncologists were responsible for 15% of prescriptions, a slight decrease compared to dasatinib. Ambulance services were involved in only 3% of cases, reflecting nilotinib's use predominantly in non‐urgent settings. Imatinib exhibited a prescribing pattern dominated by GPs (48%). Internists accounted for 27%, while haematologists/oncologists contributed again 15%. Ambulance services were responsible for 4% of prescriptions, indicating limited involvement in acute care scenarios. For bosutinib, GPs prescribed 46% of the total, followed by internists at 28%. Haematologists/oncologists accounted for 18%, while ambulance services contributed just 4%. Ponatinib exhibited a unique distribution, with the highest proportion of prescriptions (19%) attributed to haematologists/oncologists. General practitioners were responsible for 36%, the lowest among all TKIs, while internists accounted for 26%. Ambulance services represented 15% of prescriptions.

##### 3.2.1.4 Prescribed A02 Molecules Into TKI Combination

**FIGURE 4:**
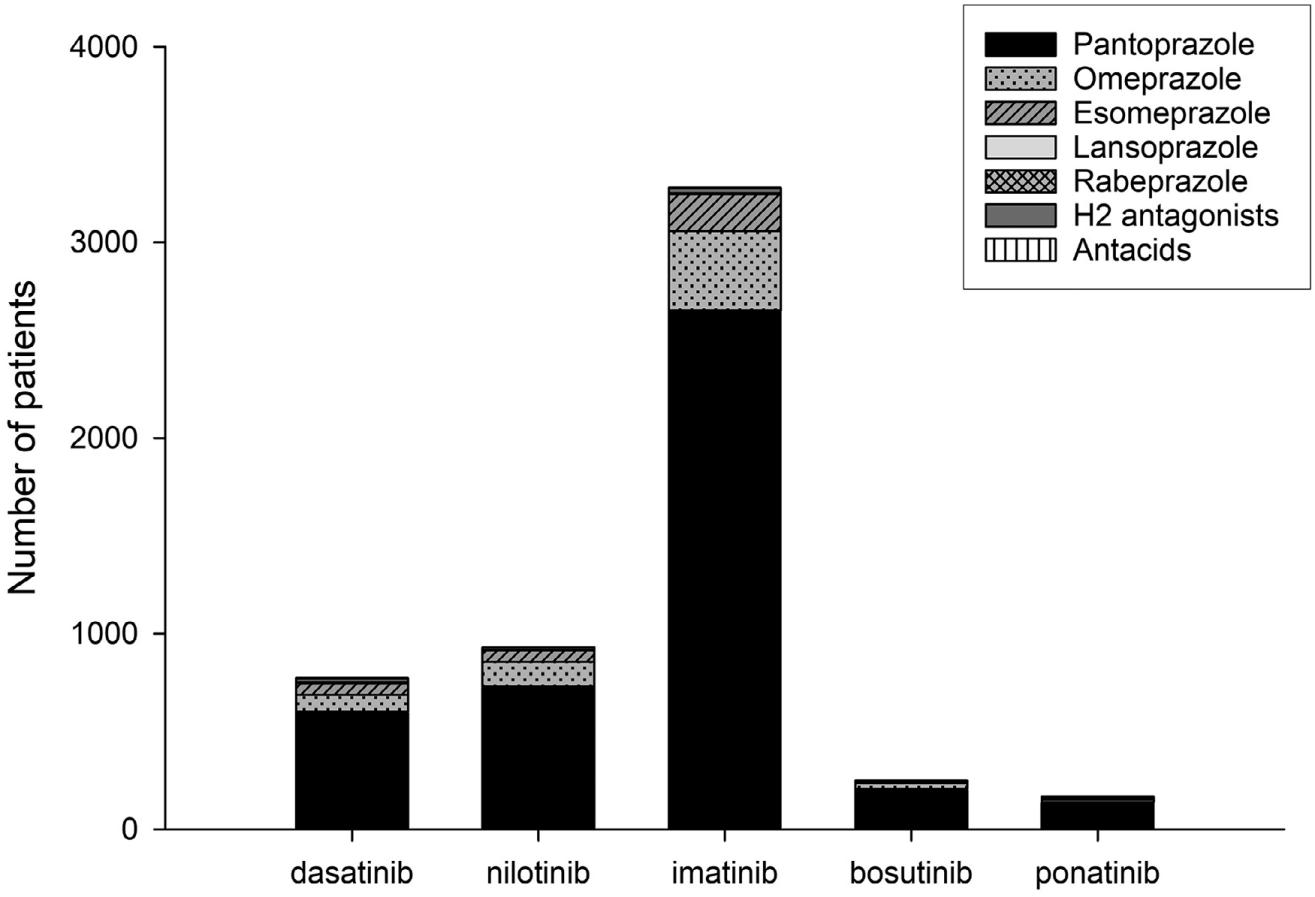
Number of patients on TKI/A02 combination based on the A02 molecule/drug class prescribed in Germany. *Source*: SHI patients, January 2024–December 2024, Patient Insights Analytics, Insight Health.

As seen in Figure 4, the prescribing patterns of gastroprotective medications, including PPIs, H2 antagonists and antacids, among physicians treating haemato‐oncology patients on TKIs reveal significant variability. This section evaluates the distribution of prescriptions for seven commonly used gastroprotective agents for each TKI.

For dasatinib, pantoprazole was the most frequently prescribed gastroprotective agent, with 601 prescriptions, followed by omeprazole (87 prescriptions) and esomeprazole (58 prescriptions). Prescriptions for lansoprazole, rabeprazole, H2 antagonists and antacids were minimal, totalling only 26 prescriptions. Nilotinib demonstrated similar trends, with pantoprazole again leading at 729 prescriptions. Omeprazole was the second most prescribed (127 prescriptions), and esomeprazole ranked third (58 prescriptions). Other agents, such as lansoprazole (2 prescriptions), antacids (1 prescription) and H2 antagonists (13 prescriptions), were rarely used, while rabeprazole was not prescribed. For imatinib, pantoprazole remained the preferred choice with 2653 prescriptions, followed by omeprazole (406 prescriptions) and esomeprazole (187 prescriptions). Other agents, including lansoprazole, rabeprazole, H2 antagonists and antacids, were minimally prescribed, accounting for just 35 prescriptions in total. Bosutinib prescribing patterns mirrored those of imatinib, with pantoprazole leading at 206 prescriptions. Omeprazole (30 prescriptions) and esomeprazole (11 prescriptions) were also used, while other agents like lansoprazole, rabeprazole and antacids were prescribed infrequently. Ponatinib had the lowest overall prescribing frequency among the TKIs, with pantoprazole being the most prescribed agent (138 prescriptions). Omeprazole (14 prescriptions) and esomeprazole (11 prescriptions) were used sparingly, and lansoprazole, rabeprazole and antacids were never prescribed. H2 antagonists were given five times.

## 4 Discussion

The impact of AS medications on the PKs of TKIs is a critical consideration in clinical practice, as seen in the literature summarized in Table S1. The data reviewed across studies demonstrate compelling evidence that AS can significantly alter the absorption and systemic exposure of these targeted therapies. However, the extent of DDIs varies depending on the specific TKI and the type of AS used. Among BCR::ABL1 TKIs in healthy volunteers, dasatinib exhibited the most pronounced reductions in PK parameters when co‐administered with PPIs and H2RAs. Specifically, omeprazole and rabeprazole led to reductions in C_max_ ranging from 38% to 96%, with corresponding AUC reductions up to 88%. Similarly, famotidine caused a 61% decrease in AUC, while aluminium/magnesium hydroxide resulted in a 55% reduction. These findings underscore dasatinib's high dependence on gastric acidity for optimal absorption, reinforcing recommendations to avoid concurrent use with PPIs [40, 42, 55, 63]. In contrast, imatinib showed no significant PK alterations when coadministered with omeprazole or antacids, indicating a lower sensitivity to changes in gastric pH [39, 50, 52]. Nilotinib demonstrated moderate decreases in C_max_ (3.4% to 27%) and AUC (8.2% to 34%), with stronger effects observed with PPIs compared to antacids or H2RAs [52, 57, 64]. For newer‐generation TKIs, bosutinib experienced a significant reduction of 39% in C_max_ and 24% in AUC when co‐administered with lansoprazole, indicating a notable DDI [37]. However, asciminib [41], ponatinib [47], futibatinib [56] and ALK inhibitors crizotinib and lorlatinib [54] showed no significant impact from AS co‐administration.

Beyond BCR::ABL1inhibitors, ALK inhibitors such as ceritinib demonstrated extreme sensitivity to gastric pH alterations, with esomeprazole reducing C_max_ by 79% and AUC by 76% [45]. Similarly, EGFR TKIs, including erlotinib [44, 53], gefitinib [34, 51], pazopanib [65] and dacomitinib [49], showed AUC reductions of 35%–47%, further emphasizing the need for caution. The HER2 inhibitor neratinib also showed a profound decrease in systemic exposure, with a 71% reduction in both C_max_ and AUC [43]. On the other hand, fedratinib [48], filgotinib [38], ripretinib [46] and cabozantinib [66, 67], crizotinib and lorlatinib [54] exhibited negligible changes in PK when administered with PPIs, highlighting their minimal dependence on gastric acidity. Surprisingly, acalabrutinib [68] demonstrated a slight increase in PK parameters with PPI co‐administration.

Interactions between TKIs and AS medications have also been studied in oncology patients (as shown in Table S2). Among BCR::ABL1TKIs, dasatinib again exhibited the most substantial reductions in systemic exposure. For instance, lansoprazole (30 mg) led to a 92.6% decrease in C_max_ compared to Phase 1 trial values, with a corresponding AUC reduction of 72.2%. Similarly, famotidine (20–40 mg) caused a reduction in AUC by 61% [60] and up to 72.2% [60]. In the category of EGFR TKIs, afatinib [53] and dacomitinib [59] exhibited minimal PK changes when co‐administered with esomeprazole and H2RAs, respectively. In contrast, erlotinib demonstrated a more pronounced DDI, with esomeprazole reducing C_max_ by 56% and AUC by 47% [53], and by 42% and 39% [61], respectively. Regorafenib seemed to be less affected by the same PPI and oncology treatments [58]. This suggests that certain EGFR TKIs may be less affected by AS medications, likely due to differences in solubility and absorption mechanisms.

The impact of AS on the efficacy and survival outcomes of TKIs in oncology patients has become a critical clinical concern, as outlined in Table S3 of this article. The data from the reviewed studies demonstrate significant variability in the effect of AS across different TKI classes and cancer types, with some drugs showing marked reductions in PFS and OS, while others remain largely unaffected. Among BCR::ABL1 TKIs, dasatinib showed the most substantial impact, with PPI co‐administration leading to a significant reduction in OS in CML patients (HR 3.5, 95% CI 2.1–5.3) [18]. In contrast, imatinib did not exhibit a statistically significant effect of PPI use on survival (HR 0.61, 95% CI 0.29–1.30 over 1 year), suggesting that imatinib's absorption and efficacy may be less influenced by changes in gastric pH [28]. For EGFR TKIs, erlotinib combined with PPIs led to a significant decrease in OS (HR 1.38, *p* = 0.003) and PFS (HR 1.71, *p* < 0.0001), suggesting that acid suppression compromises drug absorption and efficacy [10], a finding further supported in other studies [13, 17, 30, 38]. Similarly, afatinib showed reduced OS when co‐administered with PPIs (HR 1.29, *p* = 0.01) [14], while dacomitinib exhibited worsened OS outcomes in patients with extensive PPI use (HR 1.77, *p* = 0.02) [19]. However, the effect of AS on gefitinib was less consistent, with some studies indicating reduced OS or PFS [8, 12, 19, 22, 30, 69] while other papers did not [15, 27, 35, 70] likely due to different methodology. The impact of acid‐suppressive agents extended beyond TKIs targeting BCR::ABL1 and EGFR. In metastatic melanoma patients treated with dabrafenib and trametinib, PPIs were associated with significantly reduced OS (HR 2.38, *p* < 0.001) and PFS (HR 2.09, *p* < 0.001) [24]. Similarly, in RCC patients receiving sunitinib, acid suppression led to shorter OS (*p* = 0.02) and PFS (*p* = 0.04) [71], which was further supported [7]. In contrast, sunitinib‐treated patients in another study did not show significant differences in treatment duration between PPI users and non‐users, suggesting possible variations in study design or population characteristics [25, 28]. Interestingly, some TKIs, such as regorafenib [33] and CDK4/6 inhibitors (palbociclib and abemaciclib) [11, 29], did not demonstrate significant reductions in OS or PFS within DDI. Pazopanib showed various outcomes from no impact [20, 31] to partial impact [9, 23, 25]. However, in soft‐tissue sarcoma, pazopanib showed substantial reductions in both OS (HR 1.81, *p* < 0.01) and PFS (HR 1.49, *p* = 0.01) [21] and significantly reduces OS and objective response rate [72]. Overall, these findings underscore the importance of carefully considering acid‐suppressive agents co‐administration in oncology patients receiving TKIs.

Focusing on CML, the general recommendation for initial treatment is the use of TKIs, rather than cytotoxic agents, interferons, or haematopoietic cell transplantation (HCT), due to their superior balance of efficacy and toxicity [73]. Patients with CML treated with a TKI generally achieve normal or near‐normal long‐term survival, comparable to age‐ and sex‐matched control populations [74].

There is no significant difference in OS between imatinib and second‐generation (2G) TKIs (such as nilotinib, dasatinib and bosutinib) when used for the initial treatment of CML [75]. However, 2G TKIs typically induce faster and deeper remissions, and are associated with a lower risk of progression to accelerated phase (AP) or blast phase (BP), which is particularly important in patients with high‐risk chronic‐phase CML [76].

Clinicians should clearly exercise caution when prescribing acid‐suppressive (AS) medications to CML patients undergoing dasatinib therapy, as this combination may lead to suboptimal drug exposure and potentially reduce efficacy. Therapeutic drug monitoring of dasatinib plasma concentrations has been recommended to ensure adequate drug exposure and optimize treatment outcomes [77]; however, this is not common practice as it does not appear in the treatment guidelines [73]. To maintain optimal dasatinib levels, it is advised to avoid co‐administration with AS medications (for PPIs and H2RAs) or, if necessary, to space their administration by at least 2 h [40, 78, 79]. In addition, the solubility of bosutinib is pH‐dependent and absorption gets reduced when gastric pH is increased; and thus, caution should be exercised when administering bosutinib concomitantly with PPIs. Our data show that a significant percentage of both dasatinib (20%–26%) and bosutinib (30%–36%) real‐world patients take their treatment along with AS medication regardless of such warnings.

CML accounts for approximately 15%–20% of leukaemias in adults [80] with an annual incidence of 1–2 cases per 100,000, with a slight male predominance, which aligns with our dataset (in total 7171 males vs. 6032 females) [81]. The median age at presentation is approximately 50 years in clinical studies, though cancer registry data suggest it may be about 10 years older [82]. This is consistent with the age distribution observed in our dataset, particularly in patients over 50, as shown in Figure 2B. CML is rare in children [62], which is also reflected in our dataset. Our prescription data shows that imatinib, being a long‐established first‐line therapy, is widely used across all age groups. In contrast, bosutinib is primarily prescribed as a second‐line or later treatment, often for older patients, as reflected in the age distribution. Similarly, ponatinib is used mainly as a third‐line therapy for patients with more complex clinical conditions, typically seen in older age groups.

Considering PPI prescription patterns in 2018 in Germany (Bavaria), 71.2% of daily defined doses (DDDs) were prescribed by primary care physicians (315.3 million DDDs), followed by internal medicine specialists (119.1 million DDDs, 26.9%) and other physician specialities (8.7 million DDDs, 1.9%). The most common on‐label indication was gastroesophageal reflux disease (21.5%), followed by heartburn (3.4%), ulcers (2.4%) and infection with *Helicobacter pylori* (2.3%). Esophagitis and Zollinger–Ellison syndrome were very rare (0.6% and 0.01%). In users who had no documentation of an on‐label indication (*N *= 297,321), gastritis/duodenitis (24.1%) and upper stomach pain/unspecified dyspepsia (8.8%) were most frequently reported, which illustrates the fact that most of the prescribed PPIs are off‐label indications [83]. In addition, PPIs can also be obtained as over‐the‐counter medicines in most countries, which further amplifies the DDI problem.

The prescribing patterns across physician specialities from our dataset highlight the central role of GPs in managing CML patients, underscoring their key role in DDI risk awareness. Internists and haematologists/oncologists consistently contribute across all TKI prescriptions, playing an essential role in long‐term care and the management of comorbidities. The involvement of ambulance services varies, peaking with ponatinib, which is often used in acute or complex clinical scenarios. Other specialities show minimal involvement in DDIs. This observation emphasizes the importance of DDI awareness across all prescribing specialities.

Regarding gastroprotective agents, pantoprazole emerged as the most prescribed, indicating its prominent role in clinical practice in Germany. Omeprazole and esomeprazole were the next most frequently prescribed, while other agents, such as lansoprazole, rabeprazole, H2 antagonists and antacids, were used less frequently. These trends are in line with published data [84] and suggest a strong physician preference for pantoprazole, likely due to its efficacy, tolerability and compatibility with TKIs. Notably, the total patient counts in Figures 1, 2, 3, 4 are slightly different because some patients may be prescribed multiple PPIs or other A02 agents (e.g., half a year on omeprazole and half a year on pantoprazole). Therefore, such patients are counted as one patient in Figure 1 (for TKI + A02) but may be counted multiple times based on the specific agents in Figure 4. Notably, all the top ten PPI products most prescribed to TKI patients are pantoprazole packs of 100 units (data not shown), which demonstrates that the TKI/PPI co‐medication phenomenon occurs over longer periods in the therapy.

The main limitation of this study is the lack of clinical impact data. While the prescription‐based model effectively captures physician preferences and behaviours in specific indications, it does not provide insights into clinical outcomes such as hospitalizations or survival rates. These aspects could be explored in future studies. Prescription‐based models, however, are easily retrievable, patient‐blinded and ideal for analyzing physician behaviour, which was the primary goal of this paper. Another limitation is the focus on a single indication. For clarity in illustrating prescribing patterns, this study concentrated solely on TKIs used in CML. However, future research could expand to include all approved TKIs in Germany, which the research team plans to explore. Another limitation is that the database does not allow for the differentiation of indication, hence the presented patient numbers include both Ph‐positive CML patients as well as patients with Ph‐positive acute lymphoblastic leukaemia (in the case of dasatinib, imatinib and ponatinib) and other myeloproliferative diseases (imatinib). However, Ph‐positive CML is much more prevalent than Ph‐positive ALL, thus the majority of patients from this dataset are indeed CML patients. Overall, the phenomenon described in this publication is relevant to all patients taking these TKIs, regardless of indication. Considering our datasets, year‐to‐year comparisons do not indicate any particular trends in all studied molecules. Another limitation concerns the collection of prescription data, which were focused on the PPIs rather than other AS subgroups. The reasoning for this approach was the fact that the PPIs are still mostly co‐prescribed with the TKIs [83].

The decision to focus on TKIs used in CML and specific DDIs in this analysis is based on the established role of small‐molecule TKIs as the preferred treatment for newly diagnosed CML patients, with alternatives considered only when TKIs are contraindicated or poorly tolerated [18], along with the fact that some of the CML TKIs have DDI warnings or wording in their respective SmPCs (namely, dasatinib and bosutinib) while others do not. The idea was to see if SmPC warnings are sufficient to prevent the co‐prescription in the real‐world setting (Table 1).

The key takeaway from this article is the widespread prescription of TKIs, despite the potential risks of DDIs outlined in the SmPC for certain molecules. However, the extent of this variability across individual TKIs should be noted. To mitigate this, more comprehensive product labelling, information campaigns, or the development of new dosage forms could help address these concerns. For example, dasatinib formulations with reduced pH‐dependency are reported [18, 42]. Given the strong correlation between PPI use and reduced efficacy of specific TKIs, clinicians should consider alternative strategies and avoid long‐term acid suppression when possible. Future studies focusing on survival outcomes are needed to better understand these DDIs, and this is a key area of our ongoing research.

## Author Contributions

M.L. coordinated the activities of co‐authors, analyzed the prescription data, and wrote the manuscript draft. A.K. and A.N. performed the literature research and triage and co‐wrote the manuscript. J.B. designed the research, brought the original idea, collected the prescription data and revised the manuscript. C.C. performed the validation step and assisted in the discussion section. J.S. assisted in individual drafting steps and revised the manuscript.

## Funding

The authors have nothing to report.

## Ethics Statement

The authors have nothing to report.

## Consent

All consents and approvals have been obtained from the authors to publish this work.

## Conflicts of Interest

The authors declare no conflicts of interest.

## Supporting information




**Supporting File 1**: jha270279‐sup‐0001‐tableS1.docx

**Supporting File 2**: jha270279‐sup‐0002‐tableS2.docx

**Supporting File 3**: jha270279‐sup‐0003‐tableS3.docx

## Data Availability

Data is available upon request.
