# Supplementary material for: Prescription Behaviour and Drug Interactions of Acid Suppressants With Tyrosine Kinase Inhibitors in Patients With Leukaemia: Data From Germany
Source: EJHaem. 2026 Mar 28;7(2):e70279. doi: 10.1002/jha2.70279 (PMC13551348; doi:10.1002/jha2.70279)
Supplement: Supplementary file 1 [file JHA2-7-e70279-s001.docx]

Supplementary Table 3. Effect of acid suppressants on TKI Efficacy: Survival Metrics Comparison

| **ATC group** | **TKI** | **Acid-suppressant** | **Indication** | **Treatment Groups** | **No. of subjects** | **OS (HR)** | **PFS (HR)** | **Key Finding** | **Reference** |
| --- | --- | --- | --- | --- | --- | --- | --- | --- | --- |
|  |  |  |  |  |  |  |  |  |  |
| L01EA - BCR::ABL1 TKI | **Dasatinib monohydrate** | PPI: Omeprazole | CML | Dasatinib + PPI vs. Dasatinib alone | 676 | 94 % (TKI only) vs. 79 % (TKI + PPI); HR 3.5 (95% CI 2.1–5.3) | Not reported | PPI co-medication significantly reduces OS | (18) |
|  |  | PPI:  (various) | CML | Dasatinib 50mg + PPI vs. Dasatinib 50mg alone | 148 | No significant difference | Event-free survival rate 97% for Dasatinib alone versus 87% for Dasatinib + PPI (P=0.067) | Effect on EFS not statistically significant | (75) |
|  | **Imatinib** | PPIs: (various) | CML | Imatinib + PPI vs. Imatinib alone | 467 | HR 0.35 (0.07–1.71) in 90 days, HR 0.61 (0.29–1.30) in 1 year | Not reported | No significant effect of PPI use on OS | (28) |
|  |  | PPIs  H₂RAs | CML | Imatinib + PPI/H₂RA vs. Imatinib alone | 296 | NS (HR 0.74, p = 0.74) | NS (HR 0.79, p = 0.79) | Polypharmacy, including PPIs, did not significantly affect survival outcomes. | (76) |
|  | **Nilotinib** | PPIs  H₂RA | Ph+CML (in chronic phase (n = 492) and in imatinib-resistant/intolerant patients(N = 256)) | Nilotinib + PPI/H₂RA vs. Nilotinib alone | 748 | 24 months: 87 % (imatinib-resistant/intolerant patients) | Not reported | PPIs and H₂RAs did not significantly affect nilotinib efficacy. Response rates were comparable between PPI users and non-users. | (77) |
| L01EB - EGFR TKI | **Afatinib** | PPIs | EGFR-mutant NSCLC | Afatinib + PPI vs. Afatinib alone | 1248 | HR 1.29; 95% CI 1.05–1.59, p=0.01 (Afatinib: 33.2 months; Afatinib + PPI: 25.1 months) | Not reported | Significant decrease in OS | (14) |
|  |  | PPIs, H₂RAs, P-CABs | Advanced NSCLC | Afatinib + AS vs. Afatinib alone | 104 | HR 1.73 (95% CI: 0.89–3.36, p = 0.101) | Not reported | Trend towards OS reduction, NS | (30) |
|  | **Dacomitinib** | PPIs | EGFR-mutant NSCLC | Non-PPI users, PPI users, Extensive PPI users | 235 | PPI: Not reported; Extensive PPI: HR 1.77, p=0.02 | PPI: NS; Extensive PPI: HR 1.94, p=0.011 | Minimal impact on dacomitinib efficacy, though extensive PPI use associated with shorter PFS and OS | (19) |
|  | **Erlotinib** | PPIs: (various) | Lung cancer | Erlotinib + PPI vs. Erlotinib alone | 5496 | HR 1.21 (1.07–1.38) in 90 days; HR 1.11 (1.02–1.20) in 1 year | Not reported | Significantly decreased OS | (28) |
|  |  | PPIs: (various), H2RA: Ranitidine | Advanced NSCLC (Stage IIIB/IV) | Erlotinib + AS vs. Erlotinib alone | 507 | HR 1.38, p = 0.003 (Median: PPI 12.9 months vs. No PPI 16.8 months) | HR 1.71, p < 0.0001 (Median: PPI 1.4 months vs. No PPI 2.3 months) | Significantly decreased both OS and PFS | (10) |
|  |  | PPIs: Omeprazole, Pantoprazole H2RAs: Ranitidine | Advanced NSCLC | Erlotinib + AS vs. Erlotinib alone | 485 | HR 1.67, 95% CI: 1.34–2.05, p < 0.0001 (Median: AS 5.52 months vs. No AS 7.23 months) | HR 1.75, 95% CI: 1.43–2.13, p < 0.0001 (Median: AS 2.20 months vs. No AS 2.27 months) | Significant reduction in OS and PFS (combination had a higher rate of infections and diarrhea) | (13) |
|  |  | PPIs, H2RAs | Mostly lung cancer | Erlotinib + AS vs. Erlotinib alone | 76 | Erlotinib + PPI/H2RA: 24.7 months vs. Erlotinib alone: 28.5 months (p = 0.38) | Erlotinib + PPI/H2RA: 5.3 months vs. Erlotinib alone: 11.0 months (p = 0.029) | Significantly shorter PFS but no significant difference in OS or adverse events | (17) |
|  |  | PPIs, H₂RAs, P-CABs | Advanced NSCLC | Erlotinib + AS vs. Erlotinib alone | 107 | HR 1.33, 95% CI: 0.80–2.22, p = 0.275 | Not reported | No significant OS reduction | (30) |
|  | **Erlotinib, Gefitinib** | PPIs, H2RAs | EGFR-mutant NSCLC | AS users vs. AS non-users | 130 | Not reported | AS 8.7 months vs. non-AS 10.7 months (p = 0.13) | No change in OS or PFS | (35) |
|  |  | PPIs, H2RAs | EGFR-mutant and non-mutant NSCLC | AS users vs. AS non-users | 163 | Not reported | HR 2.50, 95% CI 1.61-3.88, p < 0.0001 (AS 84 days, 95% CI: 65-101 vs. non-AS 221 days, 95% CI: 125-429) | Significantly shorter PFS regardless of AS | (22) |
|  |  | PPIs, H2RAs | EGFR-mutant non-small cell lung cancer (NSCLC) | AS users vs. AS non-users | 157 | HR 1.47, 95% CI: 0.92 – 2.35, p = 0.10 (AS 11.4 months vs. non-AS 17.5 months) | HR 1.37, 95% CI: 0.89 – 2.12, p = 0.16 (AS 7.6 months vs. Non-AS: 8.7 months) | No change in OS or PFS in the overall cohort; subgroup analyses showed poorer OS/PFS in certain groups | (15) |
|  |  | PPIs, H2RAs | Advanced EGFR-mutant NSCLC | AS users vs. AS non-users | 269 | Median: Non-AS 25.0 months vs. H2RAs 15.5 months vs. PPIs 11.3 months, p = 0.002 | Median: Non-AS 11.2 months vs. H2RAs 9.4 months vs. PPIs 6.7 months, p = 0.234 (NS) | Significantly decreased OS, especially in patients with *de novo* brain metastases (OS: 11.8 vs. 16.3 months, p = 0.041) | (78) |
|  | **Erlotinib, Gefitinib, Afatinib, Osimertinib** | PPI | Lung cancer | PPI users vs. PPI non-users | 34048 | HR 1.60, 95% CI: 1.57-1.64 | Not reported | Dose-dependent increase in mortality risk | (8) |
|  | **Gefitinib** | PPI | EGFR-mutant NSCLC | Non-PPI users, PPI users, Extensive PPI users | 229 | PPI: HR 1.65, p=0.007 vs. Extensive PPI: HR 1.70, p=0.050 | NS | Shorter OS, no difference in PFS | (19) |
|  |  | H2RAs | EGFR-mutant NSCLC | H2RA users vs. H2RA non-users | 87 | H2RA: 29.6 months, 95% CI: 11.3–42.3 vs. non-H2RA: 25.5 months, 95% CI: 17.4–32.7 (p = 0.60) | HR 0.95, 95% CI: 0.60-1.48, p = 0.82 (H2RA: 8.0 months vs. non-AS: 9.0 months) | No change | (27) |
|  |  | PPIs, H₂RAs, P-CABs | Advanced NSCLC | Gefitinib + AS vs. gefitinib alone | 421 | HR 1.74, 95% CI: 1.34–2.27, p < 0.001 | Not reported | Significant OS reduction in AS users | (30) |
|  |  | PPIs (various) | EGFR-mutant NSCLC | Gefitinib + PPI vs. Gefitinib alone | 45 | NS (p = 0.779) | NS (p = 0.343) | No significant impact on survival outcomes. Plasma concentration Gefitinib correlated with longer OS and PFS | (79) |
|  |  | PPIs (various) | EGFR-mutant NSCLC | Gefitinib + PPI (high vs. low coverage) vs. gefitinib alone | 1278 | HR 1.67, p < 0.001 (Median: Non-PPI: 21.8 months vs. Low PPI: 16.7 months vs. High PPI: 13.5 months) | Not reported | Significantly decreased OS in a dose-dependent manner | (12) |
|  | **Osimertinib** | PPIs, H₂RAs, P-CABs | Advanced NSCLC | Osimertinib + AS vs. osimertinib alone | 136 | HR 5.04, 95% CI: 1.38–18.44, p = 0.015 | Not reported | Significant OS reduction in AS users | (30) |
| L01EC - BRAF inhibitors + L01EE - Mitogen-activated protein kinase (MEK) inhibition | **Dabrafenib + Trametinib** | PPIs (various) | Advanced melanoma | BRAFi/MEKi + PPI vs. BRAFi/MEKi alone | 192 | HR 2.38, p < 0.001 (Median: PPI: 24 months vs. non-PPI: Not reached) | HR 2.09, p < 0.001 (Median: PPI: 7 months vs. non-PPI: 13 months) | Significant reduction in OS and PFS | (24) |
| L01EF - Cyclin-dependent kinase (CDK) inhibitors | **Palbociclib** | PPIs (various) | HR+/HER2- ABC | Palbociclib alone vs. Palbociclib + early PPI vs. Palbociclib + long-term PPI | 486 | Not reported | Non-PPI: 28.7 months vs. E-PPI: 23.0 months (HR 1.5, 95% CI: 1.1–2.2, p=0.024) vs. LT-PPI: 23.0 months (HR 1.4, 95% CI: 1.0–1.9, p=0.035) | Poorer PFS and fewer hematological adverse events | (11) |
|  | **Palbociclib/ Abemaciclib** | PPIs (various) | Metastatic breast cancer (HR+/HER2−) | CDK4/6 inhibitor + PPI vs. CDK4/6 inhibitor alone | 112 | HR 1.23, 95% CI: 0.61-2.47, p = 0.57 (Median: PPI: 3.6 years vs. non-PPI: Not reached) | HR 1.19, 95% CI: 0.70-2.02, p = 0.53 (Median: PPI: 1.2 years vs. non-PPI: 1.3 years) | No significant difference in OS or PFS | (29) |
| L01EX - Other protein kinase inhibitors | **Axitinib, Sorafenib, Sunitinib** | PPIs (various) | mRCC | PKI + PPI, PKI alone | 2188 | HR 1.051, 95% CI 0.769-1.438, p = 0.754 (Median 24.1 months vs. 21.3 months) | HR 1.016, 95% CI 0.793-1.301, p = 0.902 (Median 5.5 months vs. 8.0 months) | No significant impact on efficacy | (16) |
|  | **Axitinib, Pazopanib, Sorafenib, Sunitinib** | PPIs | mRCC | PKI + PPI, PKI alone | 128 | Not reported | NS in time on PKI treatment (PPI: 8.7 months vs. non-PPI: 7.0 months, p = 0.41 | No significant effect on time to TKI discontinuation | (6) |
|  | **Cabozantinib** | PPIs (various) | mRCC | Cabozantinib + PPI vs. Cabozantinib alone | 99 | HR 1.44, 95% CI: 0.89–2.34, p = 0.138 (Median: PPI: 17.2 months vs. non-PPI: 22.8 months) | HR 0.84, 95% CI: 0.55–1.27, p = 0.402 (Median: PPI: 9.6 months vs. non-PPI: 9.1 months) | No significant change in OS or PFS. PPI users required more frequent dose reductions | (80) |
|  |  | PPIs (various) | mRCC | Cabozantinib + PPI vs. Cabozantinib alone | 122 | HR 1.92, p = 0.015 (Median: PPI: 15.2 months vs. non-PPI: 26.9 months) | HR 1.64, p = 0.004 (Median: PPI: 10.0 months vs. non-PPI: 13.8 months) | Significant reduction in OS and PFS | (9) |
|  |  | PPIs (various) | mRCC | Cabozantinib + PPI vs. Cabozantinib alone | 52 | Not reported | Median: PPI: 6 months vs. non-PPI: Not reached (p = 0.04 | PPIs significantly reduced PFS | (25) |
|  | **Pazopanib** | PPIs (various) | mRCC | Pazopanib + PPI vs. Pazopanib alone | 179 | HR 1.33, p = 0.158 (Median: PPI: 25.5 months vs. non-PPI: 38.5 months) | HR 1.49, p = 0.029 (Median: PPI: 9.9 months vs. non-PPI: 17.7 months) | Significant reduction on PFS, but not in OS | (9) |
|  |  | PPIs | Various cancers | Pazopanib + PPI vs. Pazopanib alone | 147 | HR 1.7, 95% CI: 1.2–2.5, p < 0.006 (PPI: 8.6 months, 95% CI: 5.9–18.6) vs. non-PPI: 17.6 months, 95% CI, 12.5–32.8) | Not reported | Significantly shorter OS and lower objective response rates | (81) |
|  |  | PPI, H2RAs | Advanced STS | AS users vs. non-AS users | 333 | HR 1.81, 95% CI: 1.31–2.49, p < 0.01 (AS: 8.0 months vs. non-AS: 12.6 months) | HR 1.49, 95% CI: 1.11–1.99, p = 0.01 (AS: 2.8 months vs. non-AS: 4.6 months) | Significant reduction in PFS and OS | (21) |
|  |  | PPI, H2RAs | mRCC | Pazopanib + AS vs. Pazopanib alone | 91 | HR 0.87, p = 0.68 (Median: AS: 27 months vs. non-AS: 23 months) | HR 0.76, p = 0.35 (Median: AS: 8 months vs. non-AS: 7 months) | No significant impact on OS or PFS | (31) |
|  |  | PPIs (various), H₂RAs: Famotidine, Ranitidine | mRCC | Pazopanib + AS vs. Pazopanib alone | 90 | HR 0.99, 95% CI: 0.51–1.93, p = 0.92 (median: AS: 28.0 months vs. non-AS: 30.1 months) | HR 1.25, 95% CI: 0.76–2.07, p = 0.85 (median: AS: 9.0 months vs. non-AS: 11.0 months) | No significant impact on OS or PFS | (20) |
|  |  | PPI: Esomeprazole; H₂RA: Famotidine | STS | Pazopanib + AS vs. Pazopanib alone | 91 | NS (p = 0.24) | HR 1.74, p = 0.044 (Median: AS: 5.3 months vs. non-AS: 6.7 months) | PFS was significantly reduced | (23) |
|  |  | PPIs (various) | mRCC | Pazopanib + PPI vs. Pazopanib alone | 166 | Not reported | Median: PPI: 11.9 months vs. non-PPI: 10.7 months (p = 0.79) | No overall PFS difference. Long-responders had significantly worse PFS | (25) |
|  | **Regorafenib** | PPIs | mCRC | PPI users vs. PPI non-users | 272 | HR 0.99, 95% CI: 0.77-1.28, p = 0.963 (PPI: 7.7 months, 95% CI: 6.6–8.8 vs. non-PPI: 6.9 months, 95% CI: 5.3–8.5) | HR 0.93, 95% CI: 0.77-1.20, p = 0.598 (PPI: 3.5 months, 95% CI: 3.0–4.0 vs. non-PPI: 3.3 months, 95% CI: 2.7–3.9) | No change in OS, TTF, or ORR | (33) |
|  | **Sorafenib** | PPIs (various) | Advanced HCC | Sorafenib + PPI vs. Sorafenib alone | 542 | HR 1.10, 95% CI: 0.82–1.41, p = 0.62 | HR 1.11, 95% CI: 0.86–1.44, p = 0.41 | No significant impact on OS or PFS | (26) |
|  | **Sorafenib, Lenvatinib, Regorafenib, Cabozantinib** | PPIs | HCC | PPI users vs. PPI non-users | 10209 | HR 1.30, 95% CI: 1.26–1.35 (1-year cumulative mortality: PPI: 71.3 %, 95% CI: 70.4–72.2 vs. non-PPI: 61.8%, 95% CI: 60.8–62.7) | Not reported | Significantly increased mortality | (32) |
|  | **Sunitinib** | PPIs (various) | RCC | Sunitinib + PPI vs. Sunitinib alone | 847 | HR 0.99, 0.66–1.49 in 90 days; HR 0.98, 0.77–1.25 in 1 year | Not reported | No significant impact on survival | (28) |
|  |  | PPIs (various) | mRCC | Sunitinib + PPI vs. Sunitinib alone | 233 | Not reported | Median: PPI: 11.3 months vs. non-PPI: 18.1 months (p = 0.15) | No significant difference in PFS | (25) |
|  |  | PPI, H2RAs | RCC | AS users vs. non-AS users | 231 | AS: 40.9 weeks, 95% CI: 26.1–74.4 vs. non-AS: 62.4 weeks, 95% CI: 42.0–82.7 (p = 0.02) | AS: 18.9 weeks, 95% CI: 11.0–23.7 vs non-AS: 23.6 weeks, 95% CI: 19.0–31.9 (p = 0.04) | Significantly shorter PFS and OS | (82) |
|  |  | PPIs (various) | mRCC | Sunitinib + PPI vs. Sunitinib alone | 557 | Median: PPI: 20.2 months vs. non-PPI: 25.7 months (p = 0.0212) | Median: PPI: 5.8 months vs. non-PPI: 8.0 months (p = 0.0912) | Significant reduction on OS, with a trend toward shorter PFS. | (7) |

Abbreviations: ATC - Anatomical Therapeutic Chemical, PPI - Proton pump inhibitor, H2RA - H2-receptor antagonist, NS - Non-significant, CML - Chronic myelogenous leukaemia, Ph+ ALL - Philadelphia chromosome-positive ALL, NSCLC - Non-small cell lung cancer, mCRC - Colorectal cancer, P-CABs - Potassium-Competitive Acid Blockers, AS - Acid suppressant, ABC - Advanced breast cancer, mRCC - Metastatic renal cell carcinoma, STS - Soft-tissue sarcoma, HCC - Hepatocellular carcinoma
