# Supplementary material for: Prescription Behaviour and Drug Interactions of Acid Suppressants With Tyrosine Kinase Inhibitors in Patients With Leukaemia: Data From Germany
Source: EJHaem. 2026 Mar 28;7(2):e70279. doi: 10.1002/jha2.70279 (PMC13551348; doi:10.1002/jha2.70279)
Supplement: Supplementary file 2 [file JHA2-7-e70279-s002.docx]

Supplementary Table 1. Pharmacokinetic drug-drug interaction studies in healthy volunteers

| **ATC group** | **TKI** | **Acid-suppressant** | **No. of subjects** | **Decrease in C_max_** | **Decrease in AUC** | **Reference** |
| --- | --- | --- | --- | --- | --- | --- |
|  |  |  |  |  |  |  |
| L01EA - BCR-ABL TKI | **Dasatinib anhydrate**  110.6 mg | PPI: Omeprazole (40 mg) | 35 | 38 % | 19 % (AUC_0-inf_), 20 % (AUC_0-t_) | (42) |
|  | **Dasatinib monohydrate** 100 mg | PPI: Omeprazole (40 mg) | 18 | 96 % | 88 % (AUC 0-24) | (62) |
|  |  | PPI: Rabeprazole (20 mg) | 10 | 92 % | 78 % (AUC_0-inf_),  84 % (AUC_0-22_) | (55) |
|  |  | H2RA: Famotidine (40 mg) | 22 | 63 % | 61 % (AUC_0-12_) | (40) |
|  |  | Antacid: Aluminium/magnesium hydroxides (30 ml) | 22 | 58 % | 55 % (AUC_0-12_) |  |
|  | **Imatinib** 400 mg | PPIs: Omeprazole (40 mg) | 12 | NS (C_max_: 2.04 mg/mL vs. 2.02 mg/mL, p = 0.97) | NS (AUC_0-inf_: 34.1 vs. 33.1 mg·h/mL, p = 0.64) | (39) |
|  |  | Antacid: Aluminium/magnesium hydroxides (20 ml) | 12 | NS (C_max_: 2.06 µg/mL vs. 2.06 µg/mL) | NS (AUC_0-inf_: 34.1 vs. 33.1 mg·h/mL, p = 0.64) | (50) |
|  |  | Antacid: Calcium carbonate (4000 mg) | 11 | NS (C_max_: 0.670 µg/mL vs. 0.618 µg/mL, p = 0.97) | NS (AUC_0-inf_: 18.4 vs. 16.9 µg·h/mL, p = 0.83) | (52) |
|  | **Nilotinib**  400 mg | PPI: Esomeprazole (40 mg) | 15 | 27 % | 34 % (AUC_0-inf_) | (63) |
|  |  | H2RA: Famotidine (40 mg) | 37 | NS (3.4 %) | NS (AUC_0-inf_: 9 %) | (57) |
|  |  | Antacid: Calcium carbonate (4000 mg) | 11 | NS (7.8 %) | NS (AUC_0-inf_: 8.2 %) | (52) |
|  | **Bosutinib** 400 mg | PPI: Lansoprazole (60 mg) | 24 | 39 % | 24 % (AUC_last_) | (37) |
|  | **Asciminib**  40 mg | PPI: Rabeprazole (20 mg) | 23 | 9 % | NS (AUC_0-inf_) | (41) |
|  | **Ponatinib**  45 mg | PPI: Lansoprazole (60 mg) | 20 | 25 % | NS (AUC_0-inf_) | (47) |
| L01EB - Epidermal growth factor receptor (EGFR) TKI | **Dacomitinib** 45 mg | PPI: Rabeprazole (40 mg) | 24 | 49.5 % | 39.2 % | (49) |
|  | **Erlotinib**  150 mg | PPI: Esomeprazole 40 mg | 9 | 56 % | 47 % (AUC_0-24h_) | (53) |
|  |  | PPI: Omeprazole 40 mg | 24 | 61 % | 46 % (AUC_0-inf_) | (44) |
|  |  | H2RA: Ranitidine 300 mg | 24 | 54 % | 33 % (AUC_0-inf_) |  |
|  |  | H2RA: Ranitidine (150 mg BID) | 24 | 17 % | 15 % (AUC_0-inf_) |  |
|  | **Gefitinib**  250 mg | PPI: Lansoprazole (15-30 mg), Esomeprazole (20 mg) | 47 | 35 % | 35 % (AUC_0-24h_) | (34) |
|  |  | H2RA: Ranitidine (450 mg BID) | 25 | 71 % | 47 % (AUC_0-inf_) | (51) |
|  | **Osimertinib** | PPI: Omeprazole (40 mg) | 47 | NS (GMR 101.65 %, 90% CI: 94.65–109.16 %) | NS (AUC0-inf GMR 106.66 %, 90% CI: 100.26–113.46 %) | (64) |
| L01ED - Anaplastic lymphoma kinase (ALK) inhibitors | **Ceritinib** 750 mg | PPI: Esomeprazole (40 mg) | 22 | 79 % | 76 % (AUC_0-inf_) | (45) |
|  | **Crizotinib** 250 mg | PPI: Esomeprazole (40 mg) | 15 | NS (C_max_: 101.84 %) | 10 % (AUC_0-inf_: 89.81 %; AUC_0-last_: 89.99 %) | (54) |
|  | **Lorlatinib** 100 mg | PPI: Rabeprazole (20 mg) | 23 | 29% | NS (AUC_0-inf_) |  |
| L01EH - Human epidermal growth factor receptor 2 (HER2) TKI | **Neratinib**  240 mg | PPI: Lansoprazole 30 mg | 15 | 71 % | 71 % (AUC 0–t),  65 % (AUC_0-inf_) | (43) |
| L01EJ - Janus-associated kinase (JAK) inhibitors | **Fedratinib** 500 mg | PPI: Pantoprazole 40 mg | 26 | NS (1.09-fold increase) | NS (AUC_0-inf_: 1.15-fold increase) | (48) |
|  | **Filgotinib** 200 mg | PPI: Omeprazole 40 mg | 13 | 27 % | NS (AUC_0-inf_) | (38) |
|  |  | H2RA: Famotidine (40 mg BID, 2h before filgotinib) | 13 | 18 % |  |  |
| L01EL - Bruton's tyrosine kinase (BTK) inhibitors | **Acalabrutinib** 100 mg | PPI: Rabeprazole (20 mg BID) | 30 | 24 % | **Increase** 14 % (AUC_0-inf_, AUC_0-last_) | (65) |
| L01EN - Fibroblast growth factor receptor (FGFR) TKI | **Futibatinib** 20 mg | PPI: Lansoprazole 60 mg | 20 | NS (C_max_ GMR: 108.4 %, 90% CI: 97.7–120.2) | NS (AUC_0-inf_ GMR: 105.2 %, 90% CI: 95.3–116.2) | (56) |
| L01EE - Mitogen-activated protein kinase (MEK) inhibitors | **Cobimetinib**  20 mg | PPI: Rabeprazole (20 mg) | 20 | NS (delayed Tmax) | NS | (66) |
| L01EX - Other protein kinase inhibitors | **Cabozantinib** 100 mg | PPI: Esomeprazole 40 mg | 21 | **Increase** 10.5 % | **Increase** 9.1 % (AUC_0-inf_) | (67) |
|  |  | PPI: Esomeprazole 40 mg | Not specified | NS (C_max_ GMR: ~108%) | NS (AUC_0-inf_ GMR: ~111%) | (68) |
|  | **Pazopanib**  800 mg | PPI: Esomeprazole 40 mg | 13 | 42 % | 40 % (AUC_0-24h_) | (69) |
|  | **Ripretinib** 50 mg | PPI: Pantoprazole 40 mg | 25 | NS (C_max_ GMR: 103 %, 90% CI: 91–117%) | NS (AUC_0-inf_ GMR: 109 %, 90% CI: 98–122%) | (46) |
|  | **Quizartinib** 30 mg | PPI: Lansoprazole 60 mg | 64 | 13.9 % | 6.0 % (AUC_0-last_), 4.7 % (AUC_0-inf_) | (70) |
|  | **Vandetanib**  300 mg | PPI: (Omeprazole 40 mg),  H2RA: (Ranitidine 150 mg) | 29 | PPI: 15 % (GMR: 85 %; 90% CI: 75–96 %)  H2RA: 8 % (GMR: 108 %; 90% CI: 96–122 %) | PPI: 6 % (GMR: 94 %; 90% CI: 89–99 %)  H2RA: 1 % (GMR: 101 %; 90% CI: 96–107 %) | (71) |

Abbreviations: ATC - Anatomical Therapeutic Chemical, PPI - Proton pump inhibitor, H2RA - H2-receptor antagonist, GMR - Geometric mean ratio, NS - Non-significant
