# Supplementary material for: Prescription Behaviour and Drug Interactions of Acid Suppressants With Tyrosine Kinase Inhibitors in Patients With Leukaemia: Data From Germany
Source: EJHaem. 2026 Mar 28;7(2):e70279. doi: 10.1002/jha2.70279 (PMC13551348; doi:10.1002/jha2.70279)
Supplement: Supplementary file 3 [file JHA2-7-e70279-s003.docx]

Supplementary Table 2. Pharmacokinetic drug-drug interaction studies in oncology patients

| **ATC group** | **TKI** | **Acid-suppressant** | **Indication** | **No. of subjects** | **Decrease in C_max_** | **Decrease in AUC** | **Reference** |
| --- | --- | --- | --- | --- | --- | --- | --- |
|  |  |  |  |  |  |  |  |
| L01EA - BCR::ABL1 TKI | **Dasatinib monohydrate** 20-100 mg | PPI: Lansoprazole (30 mg) | CML, Ph+ ALL | 5 | Not reported | 58 % | (72) |
|  |  | H2RA: Famotidine (20–40 mg) | CML, Ph+ ALL | 4 | 63 % | 61 % |  |
|  |  | H2RA: Nizotidine (300 mg) | CML, Ph+ ALL | 3 | Not reported | Not reported |  |
|  | **Dasatinib monohydrate** 100 mg QD, later 70 mg BID | H2RA: Famotidine (20 mg) | Ph+ ALL | 1 | 92.6 % | 72.2 % (AUC_0-12_) | (60) |
| L01EK - VEGFR TKI | **Axitinib**  5 mg (BID) | PPI: Rabeprazole (20 mg) | Advanced nonhematologic cancer | 6 | ↓ (no numerical data reported) | Minimal to no change (AUC_0-24h_) | (73) |
| L01EB - EGFR TKI | **Afatinib** 30–40 mg | PPI: Esomeprazole (40 mg) | Metastatic NSCLC | 18 | NS (16.2 %, p = 0.392) | NS (AUC_0-24h_: 10.2 %, p = 0.564) | (53) |
|  | **Dacomitinib** 45 mg | H2RAs: Cimetidine, Famotidine, Ranitidine, Nizatidine | NSCLC | 16 | NS | NS | (59) |
|  | **Erlotinib** 150 mg | PPI: Esomeprazole (40 mg) | Metastatic NSCLC | 9 | 56 % | 47 % (AUC_0-24h_) | (53) |
|  |  | PPI: Esomeprazole (40 mg) | Metastatic NSCLC | 28 | 42 % (p = 0.019) | 39 % (AUC_0-12h_, p = 0.004) | (61) |
|  | **Erlotinib** various doses | PPIs: Lansoprazole, Omeprazole, Esomeprazole, Rabeprazole,  H2RAs: Famotidine, Ranitidine | NSCLC | 42 | Not reported | Not reported  *C/D Ratio: (PPI ↓24 % (p < 0.05)  H2RA: ↓6 % (NS)) | (74) |
| L01EX - Other protein kinase inhibitors | **Regorafenib** 120 mg | PPI: Esomeprazole (40 mg) | Metastatic mCRC | 14 | 16.5 % (p = 0.18) | 3.9 % (AUC_0-24h_, p = 1.0) | (58) |

Abbreviations: ATC - Anatomical Therapeutic Chemical, PPI - Proton pump inhibitor, H2RA - H2-receptor antagonist, NS - Non-significant, CML - Chronic myelogenous leukaemia, Ph+ ALL - Philadelphia chromosome-positive ALL, NSCLC - Non-small cell lung cancer, mCRC - Colorectal cancer
